# Association between remnant cholesterol and arterial stiffness: A secondary analysis based on a cross‐sectional study

**DOI:** 10.1111/jch.14384

**Published:** 2021-11-09

**Authors:** Zhenwei Wang, Min Li, Jing Xie, Jing Gong, Naifeng Liu

**Affiliations:** ^1^ Department of Cardiology Zhongda Hospital School of Medicine Southeast University Nanjing China; ^2^ College of Basic Medicine and Clinical Pharmacy China Pharmaceutical University Nanjing China

## Abstract

The relationship between conventional lipid parameters and arterial stiffness (AS) has been verified by previous studies. However, it remains unknown whether non‐conventional lipid parameters have certain predictive effect on AS represented by brachial‐ankle pulse wave velocity (baPWV). Therefore, the study was to explore the relationship between remnant cholesterol (RC) and other non‐conventional lipid parameters and AS in the general population free from cardiovascular disease. The study included 912 participants aged 24–84 years from a medical health checkup center of Murakami Memorial Hospital. Logistic regression analysis and receiver operating characteristic (ROC) curves were used to examine the association between non‐conventional lipid parameters and AS. The results showed that compared with non‐AS group, the AS group had higher RC, non‐high‐density lipoprotein cholesterol (Non‐HDL‐C), atherogenic index of plasma (AIP), lipoprotein combine index (LCI), atherosclerosis index (AI), triglycerides/HDL‐C (TG/HDL‐C), Castelli's risk index I (CRI‐I) and Castelli's risk index II (CRI‐II). Then, the authors divided participants into two groups by the optimal cutoff point of 23.6 of RC determined by Youden index. The baPWV was significantly higher in higher RC group compared with lower RC group, and RC was positively correlated with baPWV. Multivariate Logistic regression analysis showed that, regarding lower RC as reference, higher RC was independently associated with higher risk of AS, independent of other risk factors (OR = 1.794, 95% CI: 1.267‐2.539, *p* = .001). The area under the curve of AS predicted by RC was higher than that of other non‐conventional lipid parameters (almost all *p* < .05). The findings indicated that increased RC was a significant predictor of AS.

## 1 INTRODUCTION

The brachial‐ankle pulse wave velocity (baPWV) is a simple indicator of the stiffness of medium and large arteries, which is simply assessed by the pressure sleeves that wrap around the limbs.^1^ A large body of evidence has been gathered to show that baPWV, a non‐invasive alternative indicator of arterial stiffness (AS), is an independent risk factor for future cardiovascular disease (CVD), and whose measurement is simple enough to make it easy to be obtained in clinical practice.^1^, ^2^, ^3^ Most studies have shown that the increase of baPWV was related to the occurrence of hypertension,^4^ diabetes,^5^ cardiac‐cerebral vascular disease and mortality.^6^, ^7^ Therefore, it is very important to identify the risk factors of baPWV. Previous studies have shown that high blood pressure and advanced age were the major risk factors for AS.^8^ Moreover, lipid metabolism is also one of the important factors leading to AS. Several studies have shown that conventional lipid parameters, including cholesterol and triglycerides, were related to AS.^9^, ^10^, ^11^ However, as far as we know, the relationship between non‐conventional lipid parameters and AS has not been reported yet, such as remnant cholesterol (RC).

Dyslipidemia is a recognized cause of CVD, which has been confirmed by many genetic and epidemiological studies.^12^, ^13^ Decreasing plasma low density lipoprotein cholesterol (LDL‐C) is a key method for the prevention of CVD, which is strongly recommended in current guidelines.^14^, ^15^ However, patients with significantly reduced LDL‐C still have a significant risk of cardiovascular residue.^16^ Recently, emerging evidence shows that RC may be responsible for the above‐mentioned residual CVD risk.^17^, ^18^ RC represents the cholesterol composition of rich in triglyceride lipoproteins, which consists of chylomicron remnants, intermediate density lipoprotein and very low‐density lipoprotein.^17^ Several studies have discussed the association between RC and CVD, metabolic diseases and major adverse cardiovascular outcomes in different people.^16^, ^19^, ^20^, ^21^, ^22^, ^23^, ^24^ Although experimental studies have demonstrated that RC promotes the formation and development of atherosclerosis through adhesion and pro‐inflammatory effects, which leads to the occurrence of related diseases,^25^, ^26^ data about the predictive implications of RC for AS derived from baPWV in the general population with different models is currently lacking.

Therefore, it is of great clinical significance to identify the relationship between RC and AS if we are to develop new therapeutic targets and to tailor risk reduction strategies that match individual risk level. Based on this, the present study was conceived with the purpose of: (1) recognizing the potential connection between RC and AS; (2) exploring whether the predictive value of RC for AS is affected by different subgroups; (3) determining whether the predictive value of RC for AS is higher than that of other non‐conventional lipid parameters.

## 2 METHODS

### 2.1 Study population

The present study is a secondary analysis based on a cross‐sectional study, which is to improve public health by early assessment of potential risk factors of subclinical disease, performed by Takuya Fukuda and coworkers at the Medical Health Checkup Center of Murakami Memorial Hospital, Gifu, Japan, from March 2004 to December 2012,^27^ the details of which have been described elsewhere.^28^ The present study included 912 participants aged 24–84 years from the above‐mentioned hospital. The original study protocol was approved by the ethics committee of Murakami Memorial Hospital, all participants of the present study provided written informed consent at the time of enrollment, and the study was consistent with the principles of Declaration of Helsinki.^27^

### 2.2 Data collection and definitions

Data of the present study were obtained from a free public database (www.Datadryad.org), which allowed researchers to download and use original data free of charge. After the authors of the original research share the data free, the raw data is protected by the data sharing policy, so we are free to use the data for secondary analysis without harming the rights and interests of the authors. When using these data, we need to follow the data sharing policy, that is, to cite data sources: Fukuda, Takuya and coworkers (2014), Data from: Association between serum γ‐glutamyltranspeptidase and atherosclerosis: a population‐based cross‐sectional study, Dryad, Dataset, https://doi.org/10.5061/dryad.m484p. In the original data file, the data variables needed for the present study are as follows: age, sex, smoking status, alcohol consumption, exercise situation, fatty liver condition, body mass index (BMI), systolic blood pressure (SBP), diastolic blood pressure (DBP), alanine aminotransferase (ALT), aspartate transaminase (AST), γ‐glutamyltranspeptidase (GGT), fasting glucose, uric acid, estimated glomerular filtration rate (eGFR), total cholesterol (TC), triglyceride (TG), low‐density lipoprotein cholesterol (LDL‐C), high‐density lipoprotein cholesterol (HDL‐C) and baPWV. The details of the collection and measurement of the above data have been described elsewhere and will not be repeated here.^27^, ^28^, ^29^

According to the original research design,^27^ smoking status was divided into two groups: none or past and current, alcohol consumption was divided into four groups: 0–40 g/week, 40–140 g/week, 140–280 g/week and more than 280 g/week, regular exercise was defined as more than once a week, and fatty liver condition derived from abdominal ultrasonography was divided into two groups: yes and no. Calculation method of BMI was: weight (kg) divided into the square of the height (meter), pulse pressure (PP) was calculated as the difference between SBP and DBP, mean blood pressure (MBP) was calculated as DBP + 0.4(SBP‐DBP),^30^ and eGFR was estimated according to the formula previously reported: eGFR = 194 × creatinine (Cr)^−1.094^ × age^−0.287^ (mL/min/1.73 m^2^) for men, and eGFR = 0.739 × 194 × Cr^−1.094^ × age^−0.287^ (mL/min/1.73 m^2^) for women.^31^

For the quantification of AS, Takuya Fukuda and coworkers used an automatic waveform analyzer from Colin Medical Technology to measure baPWV, a reliable indicator for quantifying AS, by the previously published method.^32^ First, the path lengths from the suprasternal notch to the ankle and from the suprasternal notch to the brachium were measured according to the patient's height, and the pulse wave propagation distance was calculated from the difference between the latter and the former. Then the pulse wave propagation time in this distance was calculated. Finally, baPWV was obtained by dividing the propagation distance of pulse wave by the propagation time of pulse wave, and expressed in cm/s^32^. In this study, we divided participants into two groups based on baPWV of 1400 cm/s: non‐AS (baPWV ≤ 1400 cm/s; *n* = 507) and AS (baPWV > 1400 cm/s; *n* = 405).

Non‐conventional lipid parameters were calculated according to formulas reported in previous studies, including RC, non‐HDL‑C, atherogenic index of plasma (AIP), lipoprotein combine index (LCI), atherosclerosis index (AI), TG/HDL‑C, Castelli's risk index I (CRI‐I) and Castelli's risk index II (CRI‐II). The calculation formulas of non‐conventional lipid parameters is as follows: RC = TC ‐ LDL‐C ‐ HDL‐C,^18^ Non‐HDL‑C = TC ‐ HDL‐C,^33^ AIP = log (TG/HDL‐C),^34^ LCI = TC × TG × LDL‐C/HDL‐C,^35^ AI = non‐HDL‑C/HDL‐C, CRI‐I = TC/HDL‐C, CRI‐II = LDL‐C/HDL‐C.^37^ In this study, we divided participants into two groups by the optimal cutoff point of RC determined by ROC curve analysis: lower RC (≤ 23.6; n = 376) and higher RC (> 23.6; n = 536).

### 2.3 Statistical analysis

All Statistical tests were performed with SPSS 19.0 (SPSS Inc., Chicago, Illinois, USA), MedCalc version 19.1 (MedCalc Software, Belgium) and R Programming Language (version 3.6.3). Continuous variables were expressed as mean ± standard deviation or median (quartiles: Q1, Q3) depending on whether the data was normal distribution, and the independent‐sample t‐test or Mann‐Whitney U test was used to examined the differences between groups. Categorical variables were presented as numbers (percentages), and Pearson chi‐square test or Fisher's exact test was used to tested the differences between groups. Correlations between the RC and other variables were examined by the Pearson correlation or Spearman's rank correlation test. The effect of RC on AS was evaluated by the binary Logistic regression in different models, including crude model and adjusted models. Further subgroup analyses was performed to test the consistence of the predictive significance of RC for AS according to sex, age (≤ 50 and > 50 years), smoking status, alcohol consumption, regular exercise, fatty liver, BMI (< 23 and ≥ 23 kg/m^2^), SBP (< 140 and ≥ 140 mmHg), DBP (< 90 and ≥ 90 mmHg), fasting glucose (≤ 98 and > 98 mg/dL), total cholesterol (≤ 200 and > 200 mg/dL), LDL‐C (≤ 130 and > 130 mg/dL), HDL‐C (< 40 and ≥ 40 mg/dL), and eGFR (≤ 90 and > 90 mL/min/1.73 m^2^). The model used in the subgroup analyses did not contain other covariates. Besides, possible modifications of the connection between RC and AS were also assessed by interaction tests. C‐statistics derived from receiver‐operating characteristic (ROC) curve analysis were used to test the predictive potential of non‐conventional lipid parameters for AS. DeLong's test was performed to compare the area under the curve (AUC) of each parameter. The optimal cutoff point of RC for predicting AS were determine by Youden index. A two‐tailed *p* value < .05 was regarded as statistically significant.

## 3 RESULTS

**FIGURE 1:**
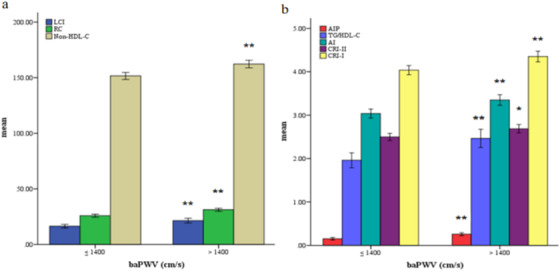
Bar graph of mean value of variables stratified by brachial‐ankle pulse wave velocity (baPWV). ** Compared with non‐AS group, participants in AS group had significantly higher LCI, RC, Non‐HDL‐C, CRII, AIP, TG/HDL‐C and AI (*p* < .001). * Compared with non‐AS group, participants in AS group had significantly higher CRI‐II (*p* < .01). Abbreviations: AS, arterial stiffness; RC, remnant cholesterol; AIP, atherogenic index of plasma; LCI, lipoprotein combine index; AI, atherosclerosis index; TG, triglycerides; HDL‐C, high‐density lipoprotein cholesterol; CRI‐I, Castelli's risk index I; CRI‐II, Castelli's risk index II

The study included 912 participants (mean age: 51.1 ± 9.6 years; 64.9% men). Baseline characteristics of the total population and participants with or without AS were showed in Table 1. Participants in AS group had significantly higher RC compared with those non‐AS group. Participants with AS had higher age and blood‐pressure parameters, and higher prevalence of fatty liver. As for laboratory parameters, participants with AS showed higher levels of fasting glucose, uric acid, AST, ALT, GGT, TG, TC and LDL‐C, while lower levels of eGFR. In terms of other non‐conventional lipid parameters, those with AS had higher levels of non‐HDL‐C, AIP, LCI, AI, TG/HDL‐C, CRI‐I and CRI‐II (Figure 1).

**TABLE 1:** Baseline characteristics of participants with and without AS

Variables	Total population (n = 912)	Non‐AS (baPWV ≤ 1400 cm/s; n = 507)	AS (baPWV > 1400 cm/s; n = 405)	*P* value
Age, years	51.1 ± 9.6	47.7 ± 9.0	55.4 ± 8.6	< .001
Sex, male, *n* (%)	592 (64.9)	314 (61.9)	278 (68.6)	.036
Smoking status, *n* (%)				.517
None or past	715(78.4)	393 (77.5)	322 (79.5)	
Current	197 (21.6)	114 (22.5)	83 (20.5)	
Alcohol consumption, *n* (%)				.086
0–40 g/week	581 (63.7)	332 (66.7)	249 (62.1)	
40–140 g/week	150 (16.4)	87 (17.5)	63 (15.7)	
140–280 g/week	90 (9.9)	45 (9.0)	45 (11.2)	
More than 280 g/week	78 (8.6)	34 (6.8)	44 (11.0)	
Regular exercise, *n* (%)	177 (19.4)	97 (19.3)	80 (20.3)	.736
Fatty liver, *n* (%)	265 (29.1)	117 (23.1)	148 (36.5)	< .001
Body mass index, kg/m^2^	23.1 ± 3.1	23.0 ± 3.3	23.3 ± 2.8	.068
Systolic BP, mm Hg	120.2 ± 15.0	114.4 ± 12.2	127.5 ± 14.9	< .001
Diastolic BP, mm Hg	76.1 ± 10.0	72.4 ± 8.4	80.8 ± 9.9	< .001
Pulse pressure, mm Hg	44.1 ± 7.4	42.0 ± 6.3	46.8 ± 7.9	< .001
Mean BP, mm Hg	93.8 ± 11.7	89.2 ± 9.6	99.5 ± 11.5	< .001
Laboratory results				
Fasting glucose, mg/dL	98.1 ± 14.1	95.8 ± 10.5	100.8 ± 17.1	< .001
Uric acid, mg/dL	5.3 ± 1.4	5.1 ± 1.4	5.4 ± 1.3	.006
eGFR, mL/min/1.73 m^2^	70.4 ± 12.0	73.0 ± 12.4	67.1 ± 10.8	< .001
AST, IU/L	19.0 (14.0, 26.0)	18.0 (14.0, 24.0)	20.0 (15.0, 28.0)	< .001
ALT, IU/L	19.0 (16.0, 23.0)	19.0 (16.0, 22.0)	20.0 (17.0, 25.0)	< .001
GGT, IU/L	19.0 (14.0, 28.0)	17.0 (12.0, 24.0)	21.0 (16.0, 31.5)	< .001
Total cholesterol, mg/dL	209.8 ± 35.9	205.6 ± 35.8	215.1 ± 35.4	< .001
Triglycerides, mg/dL	81.0 (53.0, 124.0)	72.0 (49.0, 113.0)	90.0 (62.0, 140.0)	< .001
LDL‑C, mg/dL	128.1 ± 31.7	125.7 ± 31.0	131.0 ± 32.3	.012
HDL‑C, mg/dL	53.5 ± 14.6	54.0 ± 14.0	52.9 ± 15.3	.249
RC, mg/dL	25.8 (19.1, 34.5)	23.5 (17.8, 30.9)	29.0 (21.1, 38.1)	< .001
Non‐HDL‑C, mg/dL	156.3 ± 36.2	151.5 ± 36.2	162.2 ± 35.4	< .001
AIP	0.2 ± 0.3	0.2 ± 0.3	0.3 ± 0.3	< .001
LCI	11.7 (6.2, 24.2)	10.1 (5.1, 20.5)	14.7 (7.6, 27.8)	< .001
AI	3.0 (2.3, 3.9)	2.8 (2.1, 3.7)	3.1 (2.5, 4.2)	< .001
TG/HDL‑C	1.5 (0.9, 2.7)	1.3 (0.8, 2.5)	1.7 (1.1, 3.1)	< .001
CRI‐I	4.2 ± 1.2	4.0 ± 1.2	4.3 ± 1.3	< .001
CRI‐II	2.6 ± 1.0	2.5 ± 0.9	2.7 ± 1.0	.003

*Abbreviations*: AS, arterial stiffness; baPWV, brachial‐ankle pulse wave velocity; BP, blood pressure; eGFR, estimated glomerular filtration rate; AST, aspartate transaminase; ALT, alanine aminotransferase; GGT, γ‐glutamyltranspeptidase; LDL‐C, low‐density lipoprotein cholesterol; HDL‐C, high‐density lipoprotein cholesterol; RC, remnant cholesterol; AIP, atherogenic index of plasma; LCI, lipoprotein combine index; AI, atherosclerosis index; CRI‐I, Castelli's risk index I; CRI‐II, Castelli's risk index II.

ROC curve analysis indicated that the AUC of RC for predicting AS was 0.629 (95% CI 0.597–0.661, *p* < 001). The RC of 23.6 was used as the optimal cutoff point for predicting AS. In this study, we further divided participants into two groups by the optimal cutoff point of RC: lower RC (≤ 23.6; n = 376) and higher RC (> 23.6; n = 536). Baseline characteristics of participants stratified by the RC of 23.6 were displayed in Table 2. Compared with participants in lower RC group, those with higher RC appeared to be older, display higher levels of blood‐pressure parameters, BMI and baPWV, and higher percentage of male, alcohol consumption and fatty liver. Laboratory indices including fasting glucose, uric acid, AST, ALT, GGT, TG, TC and LDL‐C were significantly higher in participants with higher RC, while HDL‐C and eGFR levels were comparatively lower. With regard to non‐conventional lipid parameters, participants with higher RC also exhibited higher non‐HDL‐C, AIP, LCI, AI, TG/HDL‐C, CRI‐I and CRI‐II compared to participants with lower RC.

**TABLE 2:** Baseline characteristics of participants stratified by the optimal cutoff point of RC

Variables	Total population (*n* = 912)	Lower RC (≤ 23.6; *n* = 376)	Higher RC (> 23.6; *n* = 536)	*P* value
Age, years	51.1 ± 9.6	49.4 ± 9.9	52.4 ± 9.2	< .001
Sex, male, *n* (%)	592 (64.9)	220 (58.5)	372 (69.4)	.001
Smoking status, *n* (%)				.191
None or past	715(78.4)	303 (80.6)	412 (76.9)	
Current	197 (21.6)	73 (19.4)	124 (23.1)	
Alcohol consumption, *n* (%)				.004
0–40 g/week	581 (63.7)	261 (70.2)	320 (60.7)	
40–140 g/week	150 (16.4)	57 (15.3)	93 (17.6)	
140–280 g/week	90 (9.9)	35 (9.4)	55 (10.4)	
More than 280 g/week	78 (8.6)	19 (5.1)	59 (11.2)	
Regular exercise, *n* (%)	177 (19.4)	78 (21.1)	99 (18.8)	.443
Fatty liver, *n* (%)	265 (29.1)	69 (18.4)	196 (36.6)	< .001
Body mass index, kg/m^2^	23.1 ± 3.1	22.4 ± 2.7	23.6 ± 3.3	< .001
Systolic BP, mm Hg	120.2 ± 15.0	116.7 ± 14.9	122.8 ± 14.5	< .001
Diastolic BP, mm Hg	76.1 ± 10.0	73.8 ± 10.0	77.8 ± 9.7	< .001
Pulse pressure, mm Hg	44.1 ± 7.4	42.9 ± 7.4	45.0 ± 7.4	< .001
Mean BP, mm Hg	93.8 ± 11.7	90.9 ± 11.6	95.8 ± 11.3	< .001
baPWV, cm/s	1415.8 ± 246.3	1358.1 ± 226.5	1456.2 ± 251.6	< .001
Laboratory results				
Fasting glucose, mg/dL	98.1 ± 14.1	94.8 ± 9.0	100.4 ± 16.4	< .001
Uric acid, mg/dL	5.3 ± 1.4	5.0 ± 1.3	5.4 ± 1.4	< .001
eGFR, mL/min/1.73 m^2^	70.4 ± 12.0	72.8 ± 13.0	68.7 ± 11.0	< .001
AST, IU/L	19.0 (14.0, 26.0)	17.0 (13.0, 22.0)	20.0 (15.0, 28.0)	< .001
ALT, IU/L	19.0 (16.0, 23.0)	18.0 (15.0, 22.0)	20.0 (17.0, 25.0)	< .001
GGT, IU/L	19.0 (14.0, 28.0)	16.0 (12.0, 22.0)	21.0 (15.0, 31.0)	< .001
Total cholesterol, mg/dL	209.8 ± 35.9	195.1 ± 31.7	220.2 ± 35.1	< .001
Triglycerides, mg/dL	81.0 (53.0, 124.0)	58.0 (41.0, 79.0)	112.0 (70.3, 157.8)	< .001
LDL‑C, mg/dL	128.1 ± 31.7	121.3 ± 29.5	132.8 ± 32.3	< .001
HDL‑C, mg/dL	53.5 ± 14.6	57.0 ± 14.5	51.1 ± 14.2	< .001
Non‐HDL‑C, mg/dL	156.3 ± 36.2	138.1 ± 30.0	169.0 ± 34.8	< .001
AIP	0.2 ± 0.3	0 ± 0.3	0.3 ± 0.3	< .001
LCI	11.7 (6.2, 24.2)	7.1 (3.8, 12.4)	19.3 (9.5, 33.4)	< .001
AI	3.0 (2.3, 3.9)	2.5 (1.9, 3.1)	3.4 (2.6, 4.4)	< .001
TG/HDL‑C	1.5 (.9, 2.7)	1.0 (.7, 1.6)	2.3 (1.3, 3.6)	< .001
CRI‐I	4.2 ± 1.2	3.6 ± 0.9	4.6 ± 1.3	< .001
CRI‐II	2.6 ± 1.0	2.3 ± 0.8	2.8 ± 1.0	< .001

*Abbreviations*: RC, remnant cholesterol; BP, blood pressure; baPWV, brachial‐ankle pulse wave velocity; eGFR, estimated glomerular filtration rate; AST, aspartate transaminase; ALT, alanine aminotransferase; GGT, γ‐glutamyltranspeptidase; LDL‐C, low‐density lipoprotein cholesterol; HDL‐C, high‐density lipoprotein cholesterol; AIP, atherogenic index of plasma; LCI, lipoprotein combine index; AI, atherosclerosis index; CRI‐I, Castelli's risk index I; CRI‐II, Castelli's risk index II.

The Pearson or Spearman's rank correlation analysis was used to examine the correlations between the RC and other variables. The RC was positively related to age, alcohol consumption, fatty liver, BMI, blood‐pressure parameters, baPWV, TG, TC, LDL‐C, fasting glucose, uric acid, AST, ALT and GGT, while negatively related to sex, HDL‐C and eGFR (Table 3).

**TABLE 3:** Correlations between the RC and other variables

Variables	Correlation coefficient	*P* value
Age	0.126	< .001
Sex	‐ 0.134	< .001
Smoking status	0.059	.073
Alcohol consumption	0.114	.001
Regular exercise	‐ 0.058	.083
Fatty liver	0.245	< .001
Body mass index	0.231	< .001
Systolic blood pressure	0.224	< .001
Diastolic blood pressure	0.235	< .001
Pulse pressure	0.136	< .001
Mean blood pressure	0.236	< .001
baPWV	0.234	< .001
Triglycerides	0.700	< .001
Total cholesterol	0.400	< .001
Low‐density lipoprotein cholesterol	0.190	< .001
High‐density lipoprotein cholesterol	‐ 0.305	< .001
Fasting glucose	0.267	< .001
Uric acid	0.227	< .001
Aspartate transaminase	0.265	< .001
Alanine aminotransferase	0.206	< .001
γ‐glutamyltranspeptidase	0.316	< .001
Estimated glomerular filtration rate	‐ 0.158	< .001

The Pearson's correlation coefficients were evaluated to detect the variables associated with the RC, including age, body mass index, systolic blood pressure, diastolic blood pressure, pulse pressure, mean blood pressure, baPWV, total cholesterol, low‐density lipoprotein cholesterol, high‐density lipoprotein cholesterol, uric acid and estimated glomerular filtration rate; The Spearman's rank correlation coefficients were evaluated to detect the variables associated with the RC, including sex, smoking status, alcohol consumption, regular exercise, fatty liver, triglycerides, fasting glucose, aspartate transaminase, alanine aminotransferase and γ‐glutamyltranspeptidase.

The RC was regarded as a continuous variable in this analysis.

*Abbreviations*: RC, remnant cholesterol; baPWV, brachial‐ankle pulse wave velocity.

**FIGURE 2:**
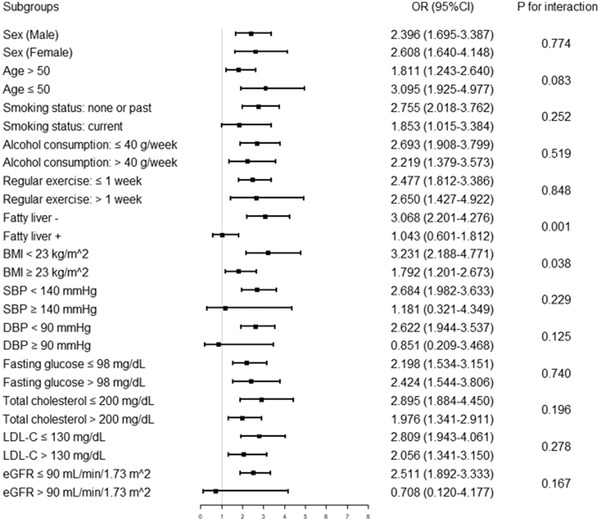
Logistic regression analysis evaluating predictive implication of RC in various stratifications. The OR was examined regarding lower RC as reference (stratified by the optimal cutoff point of RC determined by Youden index). Abbreviations: RC, remnant cholesterol; BMI, body mass index; SBP, systolic blood pressure; DBP, diastolic blood pressure; LDL‐C, low‐density lipoprotein cholesterol; HDL‐C, high‐density lipoprotein cholesterol; eGFR, estimated glomerular filtration rate; OR, odds ratio; CI, confidence interval

In Logistic regression analysis, four models (crude model and Model 1–3) including covariables with statistically significance (*p* < .1) and clinical significance were established to assess the predictive significance of RC for AS. With the increase of confounding factors, the risk of participants with higher RC developing into AS gradually decreased, while higher RC remained to be an independent risk predictor of AS, whether RC was regarded as a categorical or continuous variable (all *p* < .05 in Model 1–3) (Table 4). Further subgroup analyses was performed to test the consistence of the predictive significance of RC for AS (fourteen subgroups as mentioned above) (Figure 2). The higher RC (regarding lower RC as reference) was consistently positively correlated with AS in twelve subgroups, including sex, age, smoking status, alcohol consumption, regular exercise, fatty liver, BMI, SBP, fasting glucose, total cholesterol, LDL‐C and HDL‐C. However, in the DBP and eGFR subgroups, the correlation is opposite. Interestingly, the risk of participants with higher RC developing into AS seemed to be more noticeable in participants without fatty liver [OR (95% CI) without fatty liver 3.068 (2.201–4.276) vs. with fatty liver 1.043 (0.601–1.812), *P* for interaction = .001] and with BMI < 23 kg/m^2^ [OR (95% CI) BMI < 23 kg/m^2^ 3.231 (2.188–4.771) vs. BMI ≥ 23 kg/m^2^ 1.792 (1.201–2.673), *P* for interaction = .038].

**TABLE 4:** Predictive value of RC for arterial stiffness in different logistic regression models

	RC as a continuous variable^a^			RC as a categorical variable^b^		
	OR	95% CI	*P* value	OR	95% CI	*P* value
Crude model	1.029	1.018–1.039	< .001	2.520	1.912–3.322	< .001
Model 1	1.025	1.013–1.036	< .001	2.134	1.577–2.888	< .001
Model 2	1.020	1.008–1.032	.001	1.935	1.415–2.647	< .001
Model 3	1.018	1.006–1.031	.005	1.794	1.267–2.539	.001

Crude model: unadjusted; Model 1: adjusted for age, sex (male); Model 2: adjusted for variables included in Model 1 and smoking status, alcohol consumption, regular exercise, fatty liver; Model 3: adjusted for variables included in Model 2 and BMI, SBP, DBP, fasting glucose, uric acid, eGFR, AST, ALT, GGT, total cholesterol, triglycerides, LDL‐C, HDL‐C

*Abbreviations*: RC, remnant cholesterol; BMI, body mass index; SBP, systolic blood pressure; DBP, diastolic blood pressure; eGFR, estimated glomerular filtration rate; AST, aspartate transaminase; ALT, alanine aminotransferase; LDL‐C, low‐density lipoprotein cholesterol; HDL‐C, high‐density lipoprotein cholesterol; GGT, γ‐glutamyltranspeptidase; OR, odds ratio; CI, confidence interval.

^a^The OR was examined by per 1‐unit increase of RC.

^b^The OR was examined regarding lower RC as reference.

**FIGURE 3:**
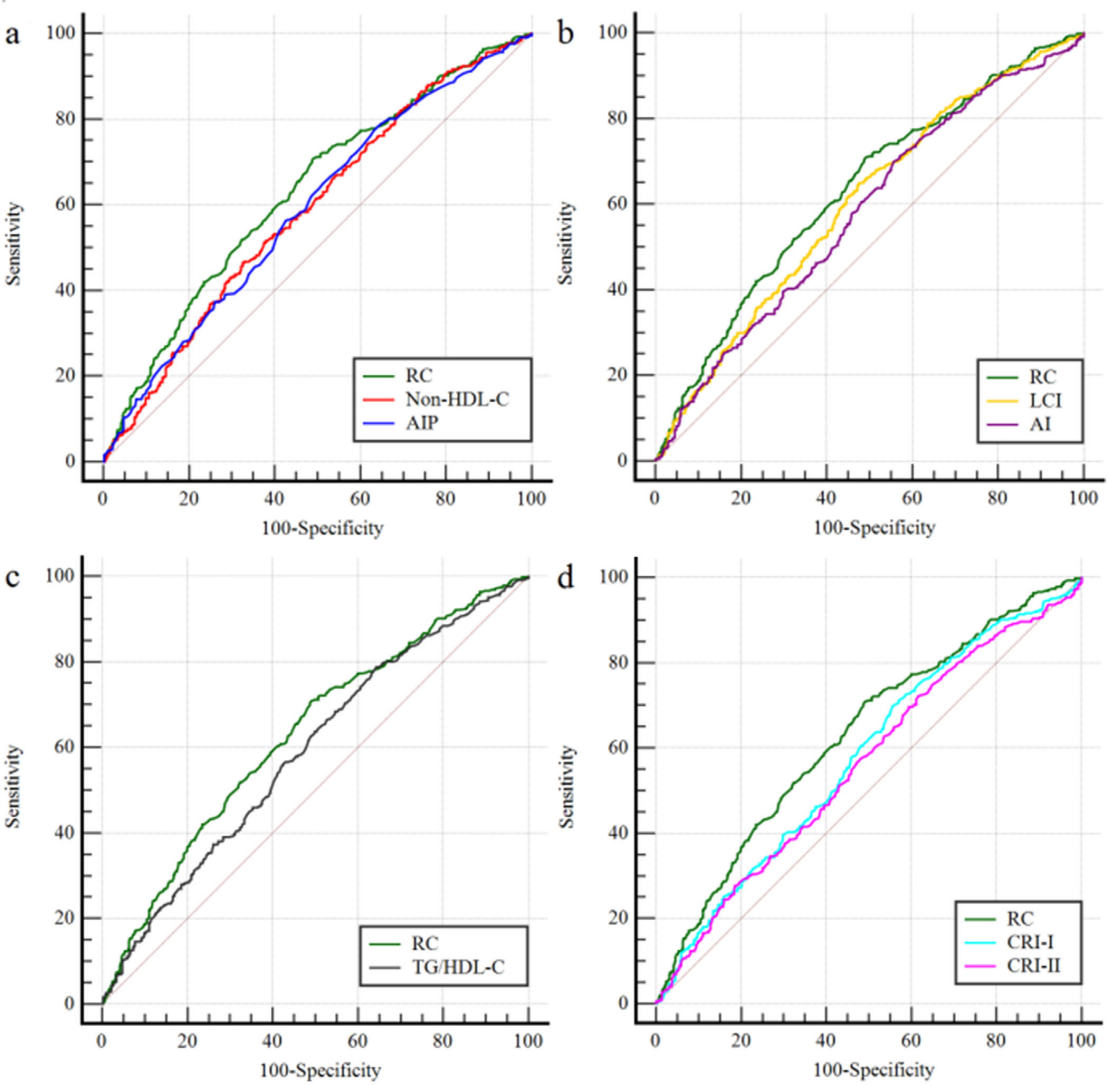
ROC curve evaluating predictive effect of RC and other non‐conventional lipid parameters for arterial stiffness. (a) RC versus Non‐HDL‐C or AIP; (b) RC versus LCI or AI; (c) RC versus TG/HDL‐C; (d) RC versus CRI‐I or CRI‐II. Abbreviations: RC, remnant cholesterol; AIP, atherogenic index of plasma; LCI, lipoprotein combine index; AI, atherosclerosis index; TG, triglycerides; HDL‐C, high‐density lipoprotein cholesterol; CRI‐I, Castelli's risk index I; CRI‐II, Castelli's risk index I

The comparative analysis of AUC of RC and other non‐conventional lipid parameters for predicting AS showed that the discriminant ability of RC was significantly higher than that of other predictive models, including non‐HDL‐C, AIP, AI, TG/HDL‐C, CRI‐I and CRI‐II (all *P* for comparison < .05). However, although the AUC of RC was larger than that of LCI, the difference was not statistically significant (*P* for comparison > .05) (Table 5, Figure 3).

**TABLE 5:** Comparison of ROC curve analysis of RC and other non‐conventional lipid parameters for the prediction of arterial stiffness

Variables	AUC	95% CI	*P* value	Z value	*P* for comparison
RC	0.629	0.597–0.661	< .001	Reference	Reference
Non‐HDL‑C	0.589	0.556–0.621	< .001	2.137	.033
AIP	0.589	0.556–0.621	< .001	2.505	.012
LCI	0.600	0.568–0.632	< .001	1.795	.073
AI	0.579	0.546–0.611	< .001	2.661	.008
TG/HDL‑C	0.589	0.556–0.621	< .001	2.513	.012
CRI‐I	0.579	0.546–0.611	< .001	2.661	.008
CRI‐II	0.560	0.527–0.593	.002	3.114	.002

*Abbreviations*: RC, remnant cholesterol; AIP, atherogenic index of plasma; LCI, lipoprotein combine index; AI, atherosclerosis index; TG, triglycerides; HDL‐C, high‐density lipoprotein cholesterol; CRI‐I, Castelli's risk index I; CRI‐II, Castelli's risk index II; AUC, area under the curve; CI, confidence interval.

## 4 DISCUSSION

As far as we know, the present study was the first report on the relationship between RC and AS. In the present study, we retrospectively explored the predictive importance of RC for AS. The main findings were as follows: (1) the RC was related to various risk factors of CVD; (2) compared with participants in lower RC group, those with higher RC showed higher levels of baPWV; (3) the higher RC (per 1‐unit increase or regarding lower RC as reference) was a strong independent risk predictor of AS in our population, although after adjusting for possible interference factors; (4) the discriminant ability of RC for predicting AS was significantly higher than that of other non‐conventional lipid parameters. These findings demonstrate that RC is independently associated with the risk of AS in the general population free from cardiovascular disease.

AS has been widely considered as an important risk factor for CVD, and it is very common in patients with CVD. Some studies have shown that AS is significantly associated with preclinical disease, development of coronary heart disease and poor prognosis.^3^, ^38^, ^39^ Therefore, for these patients, the quantitative evaluation of the degree of AS has important clinical significance for primary and secondary prevention of CVD. Percutaneous angiography has been recognized as the gold standard approach for the diagnosis of AS. However, the procedure is not only invasive, but also time‐consuming and costly, which makes it relatively difficult to apply in clinical practice. Therefore, a non‐invasive, convenient and cheap index to evaluate AS came into being, namely baPWV. A large number of studies have shown that the baPWV, as a reliable alternative quantitative marker of AS, is related to the traditional CVD risk factors including dyslipidemia and has been proved to be a encouraging biomarker for the occurrence and development of CVD.^2^, ^6^, ^40^ However, the occurrence and development of AS is inseparable from glucose and lipid metabolism and insulin resistance, of which lipid metabolism is the most important, including conventional lipid parameters and non‐conventional lipid parameters.^1^ As we all know, the one that has attracted the most attention is LDL‐C.

Raised LDL‐C is currently widely used as the primary intervention target, which is attribute to its characteristics as a major risk factor for CVD.^15^ However, when LDL‐C is reduced below the recommended level, the risk of cardiovascular events is still high, that is, the recognized residual risk, which has attracted the active attention of the majority of scholars.^26^ With the increase of attention, a growing number of studies have found that RC is responsible for the residual risk, which is mainly due to the ability of RC to converge various atherogenic effects, including the activation of monocytes, the upregulation of pro‐inflammatory factors and the increase of prothrombotic factors production.^17^, ^18^, ^25^, ^26^ From this point of view, RC seems to have the same atherogenic effect as LDL‐C, but the pathophysiological mechanism is not clear. Even so, the idea that higher RC tends to be associated with higher prevalence of AS can also be reasonably explained, as shown in this study.

In addition to basic research, many clinical studies also showed that higher RC was associated with an increased risk of CVD, independent of other traditional risk factors. Except for observational studies, genetic studies have strongly confirmed that higher RC is a fatal factor in CVD.^12^ And previous studies have shown that RC can be used not only as a prognostic index of CVD,^16^ but also as a predictor of CVD.^12^ It is also reported that RC is inextricably linked with other diseases, including aortic valve stenosis,^22^ stroke, non‐alcoholic fatty liver disease,^20^ diabetic complications^41^ and renal dysfunction.^42^ Moreover, RC is also a good predictor for subclinical CVD. As found by Lin A and coworkers and Elshazly MB and coworkers, RC is not only related to the burden of coronary atherosclerotic plaque,^43^ but also to the progression of coronary atherosclerosis.^43^ Specifically, after adjusting for corresponding confounding factors, higher RC (per 1 mmol/L increase) as a continuous variable was associated with a higher risk of plaque burden assessed by computed tomography coronary angiography (OR = 3.87, 95% CI: 1.34‐7.55, *p* = .004),^43^ and as a categorical variable, higher RC (regarding lower RC as reference) was associated with increased plaque progression assessed by intravascular ultrasonography (+ 0.53 ± 0.26 vs. ‐0.15 ± 0.25%, *p* < .001).^44^ However, the two tools mentioned above are limited in clinical application due to their invasive and expensive characteristics. Hence, we extend the risk prediction study of RC to AS, a subclinical CVD that can be assessed noninvasively, conveniently and cheaply by baPWV. Coincided with previous studies, we also found that RC was positively correlated with many traditional risk factors for CVD. In addition, this study was the first time to report that RC could independently predict AS assessed by baPWV, and it was the first time to conduct a comprehensive study on the predictive effect of non‐conventional lipid parameters on AS. It was found that RC had a greater recognition of AS than other non‐conventional lipid parameters. However, in the subgroup analysis, to our surprise, the predictive power of RC for AS was significantly reduced or even reversed in people with fatty liver and BMI ≥ 23 kg/m^2^, suggesting that fatty liver and higher BMI might interfere with the independent predictive effect of RC on AS. These two groups will be the focus of our attention in the future.

It is worth noting that there is no unified definition of RC at present, previous study have shown that RC represents the cholesterol composition of rich in triglyceride lipoproteins, which consists of chylomicron remnants, intermediate density lipoprotein and very low‐density lipoprotein.^17^ However, these indicators are not routinely measured in clinical practice, which makes them not suitable for popularization and application. Gven this, RC, which is derived from the commonly used clinical indicators (TC, LDL‐C and HDL‐C), has been proposed and widely used, and the RC in this study was calculated according to this accepted formula.^18^ Although some studies have suggested that non‐HDL‐C can be used as an alternative index of RC,^45^ and previous studies have shown that non‐HDL‐C can be used as an independent predictor of CVD,^33^, ^36^ the non‐HDL‐C level obtained in this study is not only much higher than the calculated RC, but also the predictive value of AS is lower than RC. Therefore, the calculated RC is sufficient to meet the needs of clinical management and scientific research. In addition to RC and non‐HDL‐C, previous studies have also shown that AIP, AI, LCI, CRI‐I, CRI‐II and TG/HDL‐C are associated with cardiovascular disease,^11^, ^46^, ^47^, ^48^, ^49^ but we found that the predictive performance of these non‐conventional lipid parameters is lower than that of RC. In view of this, RC seems to be the most important atherogenic indicator besides LDL‐C. Nowadays, with the development of precision and targeted therapy, accurate measurement of each pathogenic component is very important for disease management. A large clinical trial has reported that intensive lipid‐lowering therapy in patients with higher RC can reduce the risk of CVD.^50^ Taken together, these data suggest that RC may be both a prognostic marker and a potential target for future therapeutic interventions.

Innovatively, our findings added to the evidence of RC and CVD from clinical to subclinical diseases. Moreover, we comprehensively integrated different forms of non‐conventional lipid parameters for the first time and compared their predictive value for AS. Therefore, this study provided additional information that the estimation of RC and other non‐conventional lipid parameters may be of clinical significance in primary prevention to identify people at risk of CVD. In spite of this, several limitations still existed in this study. Firstly, the present study was a secondary analysis based on a cross‐sectional study, so it was impossible to obtain all the original data. Secondly, the present study was also a cross‐sectional study, which could not identify the causal relationship between RC and AS. Moreover, in Logistic regression analysis, we only controlled for several meaningful confounding factors, but there might be other confounding factors not included in our study, such as inflammatory indicators and medication history including anti‐dyslipidemic and anti‐hypertensive medications. Besides, dichotomizing baPWV may lead to several problems, such as the loss of information, the increase of potential confounding factors, the decrease of statistical ability and the bias of results. Furthermore, in this study, defining baPWV > 1400 cm/s as an indicator of higher AS may overestimate the prevalence of AS. Finally, The data of this study only came from the general population of a single ethnic group, so the findings may not be applicable more populations broadly.

## 5 CONCLUSIONS

Taken together, this is the first study to demonstrate that higher RC was a independent predictive factor for participants with AS, which might enrich the research field of predictors of AS, introduce an available indicator for risk management of AS and provide new insights on the necessity of monitoring RC in participants with impaired lipid metabolism for CAD risk assessment. Further prospective and randomized studies need to be conducted to determine whether interventions for RC have a positive effect on improving AS.

## CONFLICT OF INTEREST

The authors have no conflicts of interest to disclose.

## AUTHOR CONTRIBUTIONS

Zhenwei Wang designed the study, collected and analyzed the statistics, and wrote the manuscript. Min Li involved in study conception, data analysis, writing and revising the manuscript. Jing Xie and Jing Gong made contribution to the writing and performed statistical analysis. Naifeng Liu coordinated and supervised data collection, and reviewed the manuscript.
